# Targeting Surveillance for Zoonotic Virus Discovery

**DOI:** 10.3201/eid1905.121042

**Published:** 2013-05

**Authors:** Jordan Levinson, Tiffany L. Bogich, Kevin J. Olival, Jonathan H. Epstein, Christine K. Johnson, William Karesh, Peter Daszak

**Affiliations:** EcoHealth Alliance, New York, New York, USA (J. Levinson, T.L. Bogich, K.J. Olival, J.H. Epstein, W. Karesh, P. Daszak);; National Institutes of Health, Bethesda, Maryland, USA (T.L. Bogich);; Princeton University, Princeton, New Jersey, USA (T.L. Bogich);; School of Veterinary Medicine, University of California, Davis, California, USA (C.K. Johnson)

**Keywords:** wildlife, mammals, zoonoses, virus discovery, surveillance, pathogen, reservoir host, viruses, ecohealth

## Abstract

We analyzed a database of mammal–virus associations to ask whether surveillance targeting diseased animals is the best strategy to identify potentially zoonotic pathogens. Although a mixed healthy and diseased animal surveillance strategy is generally best, surveillance of apparently healthy animals would likely maximize zoonotic virus discovery potential for bats and rodents.

Nearly two thirds of emerging infectious diseases that affect humans are zoonotic, and three fourths of these originate in wildlife, making surveillance of wildlife for novel pathogens part of a logical strategy to prevent the future emergence of zoonoses (*1*–*4*). Wildlife are thought to harbor a high diversity of unknown pathogens, but global characterization of this diversity would be costly and logistically challenging (*5*). Given limited resources for pandemic prevention, there is public health benefit in focusing pathogen discovery on those species most likely to harbor novel zoonoses (*3*,*4*). 

One strategy to maximize the likelihood of discovering novel pathogens is surveillance of animal die-offs, outbreaks in wildlife, or diseased wildlife. We analyzed a database of known zoonotic viruses in mammal hosts to answer the driving question of whether we should stratify surveillance strategies (i.e., conduct surveillance of visibly diseased vs. apparently healthy animals) by wildlife host groups to best detect novel pathogens with zoonotic potential. In answering this question, we can better determine how host and virus taxonomy might influence our decisions about applying limited surveillance resources to a growing global health problem.

## Methods

We focused our analysis on mammalian hosts and viruses because they, more than any other host–pathogen type, are likely to be associated with emerging infectious diseases of humans (*3*,*6*). We constructed a database of all emerging viruses of humans that were previously identified as originating in wildlife; the database was supplemented with all zoonotic viruses with nonhuman mammalian hosts found in the International Committee on the Taxonomy of Viruses database (www.ictvdb.org) (*2*). For each zoonotic virus, we conducted a literature search for reports of infection in any mammalian host, using the virus name and relevant synonyms (www.ictvdb.org) as keywords in Web of Knowledge (http://wokinfo.com/), Wildlife Disease Association meeting abstracts (http://wildlifedisease.org/wda/CONFERENCES.aspx), Google Scholar (http://scholar.google.com/), and the Global Mammal Parasites Database (www.mammalparasites.org). The resulting 605 host–pathogen relationships included 56 unique viruses classified in 17 taxonomic families and 325 unique mammals classified in 15 taxonomic orders. We excluded rabies from our analysis because the intense research effort on this virus and its high pathogenicity in almost all of its wide range of hosts (*7*) would skew the data disproportionately.

We then conducted a secondary literature search to determine whether viruses in our database cause signs of disease in their wildlife hosts. For the search, we used an aggregate of all publications available in PubMed (www.ncbi.nlm.nih.gov/pubmed/), Web of Science (http://thomsonreuters.com/products_services/science/science_products/a-z/web_of_science/), BIOSIS Previews (http://thomsonreuters.com/products_services/science/science_products/a-z/biosis_previews/), and Biologic & Agricultural Index Plus (www.ebscohost.com/academic/biological-agricultural-index-plus); search terms consisted of virus names and International Committee on the Taxonomy of Viruses synonyms, host genus and species names, and common names [reconciled to the 2005 version of Mammal Species of the World (*8*)]. All resulting abstracts and available full text reports were examined until the first robust report of visible disease was encountered. Viruses were identified as causing visible disease in a host if individual or epizootic death or grossly visible or otherwise observable signs of illness (e.g., high fever, loss of mobility, or severe decline in body condition) were reported. A report was considered robust only if infections were confirmed by PCR analysis or virus isolation and clinical signs were explicitly recorded to have occurred during active infection. We excluded studies reporting only serologic findings because of potential cross-reactivity among related viruses and poor correlation between serologic status and concurrent infection. Our criteria of stopping a search once any evidence for visible disease was found meant that for mammal–virus pairs without visible disease, the search was exhaustive.

We considered diseases to be nonpathogenic in their hosts only if actively infected animals were explicitly reported to be free of visible disease. Animals with less clear signs of disease, such as nasal discharge or death of neonates, were not considered asymptomatic because of the low detection probability associated with these traits in wild mammal surveillance. We rejected reports of experimentally induced disease because of the risk that dosage and inoculation technique would not be consistent with naturally occurring infections. However, we included experimental studies if actively infected animals remained asymptomatic, with the assumption that 1) clinical signs of infection were most likely to be seen in animals monitored in laboratory settings than in the wild and 2) stressful conditions in captivity would heighten the likelihood of a normally benign pathogen leading to clinical signs (*9*). Furthermore, compared with naturally occurring infections, experimental infections often involve more direct routes of inoculation and are therefore more likely to induce disease.

## Analyses

We conducted a logistic regression analysis, using Firth’s bias reduction procedure (*10*) as used by the brglm (bias reduction in generalized linear models) package of R v2.15-2 (http://cran.r-project.org/bin/windows/base/), of apparent host disease as a function of host taxonomy and virus taxonomy for the subset of mammal–virus pairs for which the host order or virus family had at least 3 records in the database. We then calculated odds ratios for each host taxonomic order and virus family relative to the reference categories (Artiodactyla and *Flaviviridae*) and the predicted probability of being symptomatic for all species order–virus family combinations.

## Results

**Figure 1:**
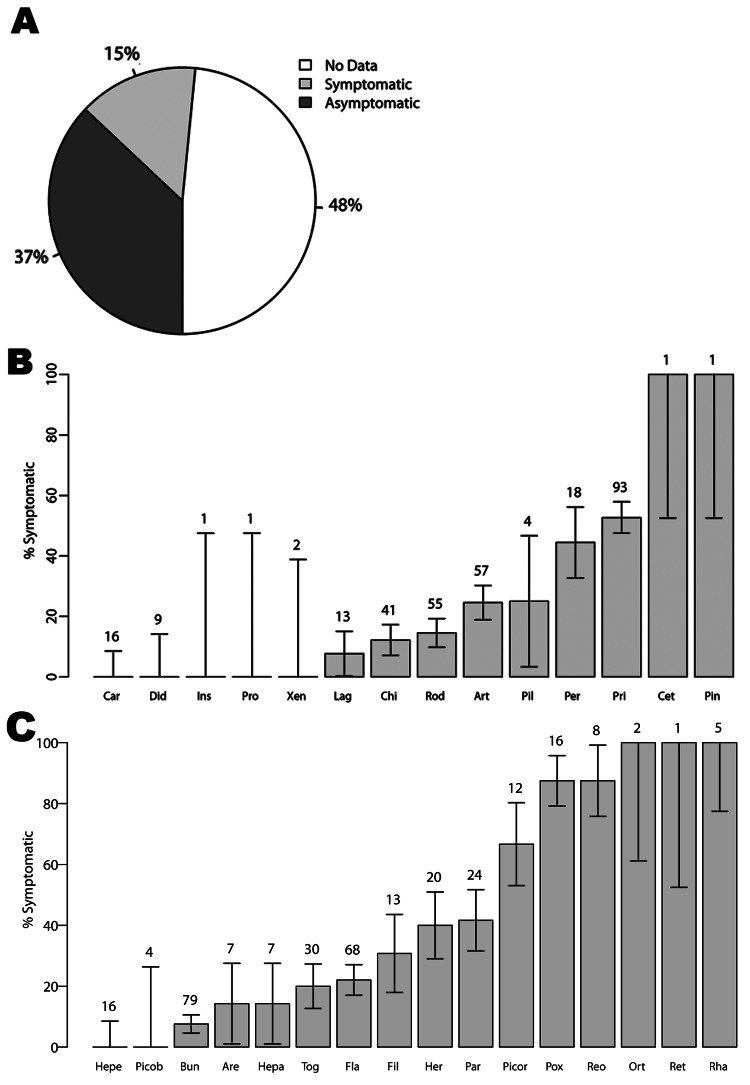
A) Percentage of host–virus pair reports describing symptomatic (observable) disease, asymptomatic disease (no observable disease), or no data (no description of disease included). B) Percentage of symptomatic hosts by mammal taxonomic order. C) Percentage of viruses, by taxonomic family, for which hosts are reported symptomatic. SEs (error bars) were calculated assuming binomial error structure. The total number of each host order or virus family included in the database is given above each bar. All host orders and virus families in the database are included here, but analyses are limited to those host orders or virus families with at least 3 entries in the database. See the online Technical Appendix (wwwnc.cdc.gov/EID/article/19/5/12-1042-Techapp1.xlsx) for the full database of host–virus pairs and disease states.

Our search of the 605 mammal–virus associations investigated yielded explicit information on host health in 52% of the 312 mammal–virus pairs. Of these, ≈28% (n = 88) of infected wildlife hosts were reported to have had visible disease and 72% (n = 224) were reported without evidence of visible disease (Figure 1, panel A). The proportion of hosts that were symptomatic differed across host order (Figure 1, panel B) and virus family (Figure 1, panel C).

We found that virus family and host order were significant predictors of disease status (χ^2^ = 88.70, p<0.001 and χ^2^ = 59.45, p<0.001, respectively). Species infected with paramyxoviruses, poxviruses, and reoviruses were more likely to have visible disease (p = 0.02, p = 0.001, and p = 0.04, respectively), and species infected with bunyaviruses were less likely to have visible disease relative to the reference category (p = 0.01) (Table). Hosts infected with filoviruses were marginally more likely to have visible disease (p = 0.08) (Table).

**Table:** Logistic regression analysis with bias reduction of whether a host presents with disease for 234 mammal–virus pairs from 5 taxonomic orders of mammals and 10 taxonomic families of viruses*

Predictor†	Values for categorical predictors relative to level of reference category					
	Coefficient	SE	Z test statistic	p value	Odds ratio	95% CI
Constant	–0.33	0.58	–0.56	0.58	0.72	0.23–2.26
Virus family (reference category: *Flaviviridae*)						
* Bunyaviridae*	–1.74	0.64	–2.71	0.01	0.18	0.05–0.62
* Filoviridae*	3.26	1.83	1.78	0.08	26.07	0.72–944.49
* Herpesviridae*	0.10	0.65	0.16	0.87	1.11	0.31–3.94
* Paramyxoviridae*	3.43	1.42	2.41	0.02	30.95	1.90–503.52
* Picornaviridae*	1.12	0.76	1.48	0.14	3.08	0.69–13.68
* Poxviridae*	2.29	0.81	2.82	<0.001	9.90	2.01–48.72
* Reoviridae*	2.13	1.05	2.02	0.04	8.39	1.07–66.12
* Rhabdoviridae*	9.20	2.39	3.85	<0.001	‡	‡
* Togaviridae*	–0.36	0.63	−–0.58	0.56	0.70	0.20–2.38
Species order (reference category: Artiodactyla)						
Chiroptera	–6.47	1.81	–3.57	<0.001	0.00	0–0.05
Perissodactyla	0.58	0.76	0.77	0.44	1.79	0.40–8.03
Primates	–0.16	0.68	–0.24	0.81	0.85	0.22–3.24
Rodentia	–1.12	0.67	–1.66	0.10	0.33	0.09–1.22

*The subset of data used was selected by using a cutoff of at least 3 records in the database to avoid making inference about host orders or virus families, for which we had very little information. †Virus and host reference groups were selected as those for which sample size was sufficiently large and symptomatic infection was moderate (see Figure 1). ‡All host–virus pairs were symptomatic.

**Figure 2:**
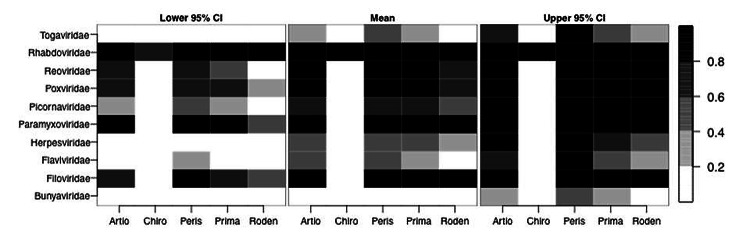
Probability of being symptomatic as determined by logistic regression analysis, with bias reduction of whether a host is diseased, for 234 mammal–virus pairs. Pairs represent mammals from 5 taxonomic orders and viruses from 10 taxonomic families. Probabilities are based on the predicted values of the logistic regression and are given on a 5-point gray scale (key on right). CIs were calculated asthe coefficient plus 1.96 × SE (from Table). See Table for detailed results of the regression analysis.

Relative to the reference category, species classified in the order Chiroptera were less likely to have visible disease (p<0.001), and species in the order Rodentia were marginally less likely to have visible disease (p = 0.10) (Table). Compared with species in other orders, species in the order Chiroptera had a lower probability of visible disease (Figure 2), although all Chiroptera species infected with nonrabies rhabdoviruses had a high probability of visible disease. In the dataset, all host–pairs infected with rhabdoviruses were in the order Chiroptera and were reported with visible disease in that host (Figure 1).

## Discussion

Nonhuman primates (*11**–**13*) and species classified within the taxonomic orders Chiroptera and Rodentia are the primary mammals targeted for zoonotic disease surveillance. Our data suggest that species in the orders Chiroptera and Rodentia are less likely than species in other orders to have visible disease (Figure 1). The mechanism behind this relationship is a critical area for additional research. In general, we found that the probability of having visible disease depends on the taxonomic classification of the host and virus, and Chiroptera is the only host order for which a single strategy (in this case, healthy animal surveillance) can be applied across nearly all virus families, excluding *Rhabdoviridae*. Therefore, particularly for the case of novel virus detection, our results point to a mixed strategy of targeted syndromic and healthy animal surveillance across host and virus taxonomies. A mixed strategy could combine apparently healthy animal surveillance (particularly in Chiroptera) with syndromic surveillance in other wildlife and domestic animal hosts. Syndromic surveillance has proven useful where secondary animal hosts are involved [e.g., surveillance for West Nile virus (*14*), henipaviruses (*15*,*16*), and Ebola virus (*17*)].

There are limitations to our study, particularly ascertainment and reporting biases, as acknowledged in previous studies of emerging infectious diseases (*2*,*3*). In addition, differences in the number of species belonging to each order, the difficulty of testing inaccessible species, and limits to reliable diagnoses of emerging viruses have an effect, especially in resource-poor settings. Furthermore, many disease states are not recognizable in free-ranging mammalian species under field conditions. Last, there is a risk that an animal may be co-infected with several agents, only one of which causes disease; that co-infection may have an additive or synergistic effect on clinical signs; and that anthropozoonotic viruses artificially inflate the disease count of mammals in some taxonomic orders over others. However, our findings were determined on the basis of an aggregation of the best data available on host health as it relates to zoonotic viruses, and they have useful implications for public health.

Our analysis supports a holistic, probability-based approach to zoonotic virus discovery, specifically, continued analysis of passively and actively reported deaths and increased investment in broad surveillance of healthy wildlife. The latter could be targeted geographically to those regions most likely to generate novel emerging infectious diseases (*2*) or taxonomically to groups that are reservoirs for the highest proportion of zoonoses (*3*,*18*). These efforts could be envisaged as part of a strategy for smart surveillance, heightening the opportunity for discovery of novel zoonoses, particularly if wildlife are sampled at key interfaces where contact with human or domestic animals (and thus the opportunity for spillover) is highest.

Technical Appendix The full database of host–virus pairs and disease state.
